# Multi-omics analyses of the heterogenous immune microenvironment in triple-negative breast cancer implicate UQCRFS1 potentiates tumor progression

**DOI:** 10.1186/s40164-025-00672-1

**Published:** 2025-06-16

**Authors:** Yuhui Tang, Aiqi Xu, Zhongbiao Xu, Jindong Xie, Wei Huang, Liulu Zhang, Yitian Chen, Lu Yang, Shasha Du, Kun Wang

**Affiliations:** 1 State Key Laboratory of Oncology in South China, Guangdong Provincial Clinical Research Center for Cancer, Sun Yat-Sen University Cancer Center, Guangzhou, 510060 People’s Republic of China; 2 Department of Breast Cancer, Cancer Center, Guangdong Provincial People’s Hospital (Guangdong Academy of Medical Sciences), Southern Medical University, Guangzhou, 510080 People’s Republic of China; 3 Department of Radiotherapy, Cancer Center, Guangdong Provincial People’s Hospital (Guangdong Academy of Medical Sciences), Southern Medical University, Guangzhou, 510080 People’s Republic of China

**Keywords:** Triple-negative breast cancer, Multi-omics, Tumor immune microenvironment, IL32^high^ Treg, UQCRFS1, Immunotherapy resistance

## Abstract

**Background:**

Triple-negative breast cancer (TNBC) is commonly characterized by high-grade and aggressive features, resulting in an augmented likelihood of distant metastasis and inferior prognosis for patients. Tumor immune microenvironment (TME) has been recently considered to be tightly correlated with tumor progression and immunotherapy response. However, the actual heterogenous TME within TNBC remains more explorations.

**Methods:**

The thorough analyses of different cell types within TME were conducted on the self-tested single-cell RNA sequencing dataset which contained nine TNBC treatment-naïve patients, including subclusters classification, CellChat algorithm, transcription factors (TFs) expression, pseudotime analysis and functional enrichment assay. The malignant epithelial cluster was confirmed by copy number variations analysis, and subsequently LASSO-Cox regression was carried out to establish a Malignant Cell Index (MCI) model on the basis of five crucial genes (BGN, SDC1, IMPDH2, SPINT1, and UQCRFS1), which was validated in several TNBC cohorts through Kaplan–Meier survival and immunotherapy response analyses. The public spatial transcriptome, proteome data and qRT-PCR, western blotting experiments were exploited to corroborate UQCRFS1 expression in RNA and protein levels. Additionally, functional experiments were implemented to unravel the impacts of UQCRFS1 on TNBC cells.

**Results:**

The diverse subclusters of TME cells within TNBC were clarified to display distinct characteristics in cell–cell interactions, TFs expression, differentiation trajectory and functional pathways. Particularly, IL32^high^ Treg imparted an essential effect on tumor evasion and predicted a worsened prognosis of TNBC patients. Furthermore, MCI model enabled to notify the inferior prognosis and immunotherapy resistance in TNBC. Ultimately, UQCRFS1 knockdown dampened the proliferative and migratory competence in vitro as well as tumor growth in vivo of TNBC cells.

**Conclusions:**

Our study offers innovative perspectives on comprehending the heterogeneity within TME of TNBC, thereby facilitating the elucidation of TNBC biology and providing clinical recommendations for TNBC patients' prognosis, such as IL32^high^ Treg infiltration, MCI evaluation, and UQCRFS1 expression.

**Supplementary Information:**

The online version contains supplementary material available at 10.1186/s40164-025-00672-1.

## Introduction

Breast cancer (BC) demonstrates a significant degree of molecular phenotype heterogeneity, which renders it to be one of the most prevalent malignancies globally [1]. Among the subtypes of BC, triple-negative breast cancer (TNBC) is featured by the lack of expression of hormone receptor (HR) and human epidermal growth factor receptor-2 (HER2) and is essentially correlated to the relatively impaired survival and also necessitates the employment of other molecular markers for prognostication and treatment response evaluation [2]. In recent decades, there have been notable advancements in comprehending the molecular heterogeneity of TNBC [3–8]. These studies offer comprehensive understandings of the molecular phenotypes of TNBC and depict the effective alternatives. Nonetheless, the most of biological mechanisms underlying the unfavorable outcomes of TNBC remain elusive. Consequently, it is indispensable to continue to exploring the molecular attributes associated with TNBC.

The investigations of TNBC progression through bulk transcriptomics are hindered by several factors, such as copy number variation (CNV) and the misleading infiltration by non-tumoral cells, which complicate their results. The comprehension of the correlation between the tumor immune microenvironment (TME) and malignant cells relies on the precise characterization and verification of distinct cellular states. Furthermore, the presence of intra-tumorigenic heterogeneity serves as a pivotal role in determining the prognosis and aggressiveness of cancer [9, 10]. Besides, the considerable level of intra-tumor heterogeneity observed among TNBC cells introduces significant challenges in accurately discerning genetic diversity through bulk mRNA sequencing, thereby rendering it a subject of intense controversy. The previous identifications of potential diagnostic markers and therapeutic targets primarily were relied on the bulk profiling technologies, without considering the intra-tumoral heterogeneity, thereby limiting their applicability to all patients.

Single-cell RNA sequencing (scRNA-seq) technology sheds a light on both intrinsic and extrinsic features of cancerous cells in an accurate way, which is expected to facilitate the resolution of the issue. It possesses the ability to discern distinct cellular subsets, depict clonal diversity, and notably ascertain the pivotal factor that influences tumor heterogeneity [11–14]. Moreover, the verifications of cancer subtypes enable the assessment of patients' treatment responses and the evaluation of improvements in clinical outcomes [15–17]. Recent advances in other omics like proteomics and spatial transcriptome (ST) also provide brand new perspectives of tumor heterogeneity. The primary objective of proteomics is to confirm and describe the operational proteins responsible for the progression of malignancy. Additionally, proteomics endeavors to uncover biomarkers that can enhance the early diagnosis of cancer, prognosticate outcomes, evaluate the effectiveness of therapeutic interventions, identify innovative targets for drug development, and ultimately establish personalized medical approaches [18, 19]. ST technique enables the systematic measurement of expression levels of all or a majority of genes within tissue space, and has been widely utilized to generate valuable biological insights in numerous areas [20–22]. Hence, it is imperative to apply multi-omics methods to uncover molecular attributes associated with TNBC.

**Fig. 1:**
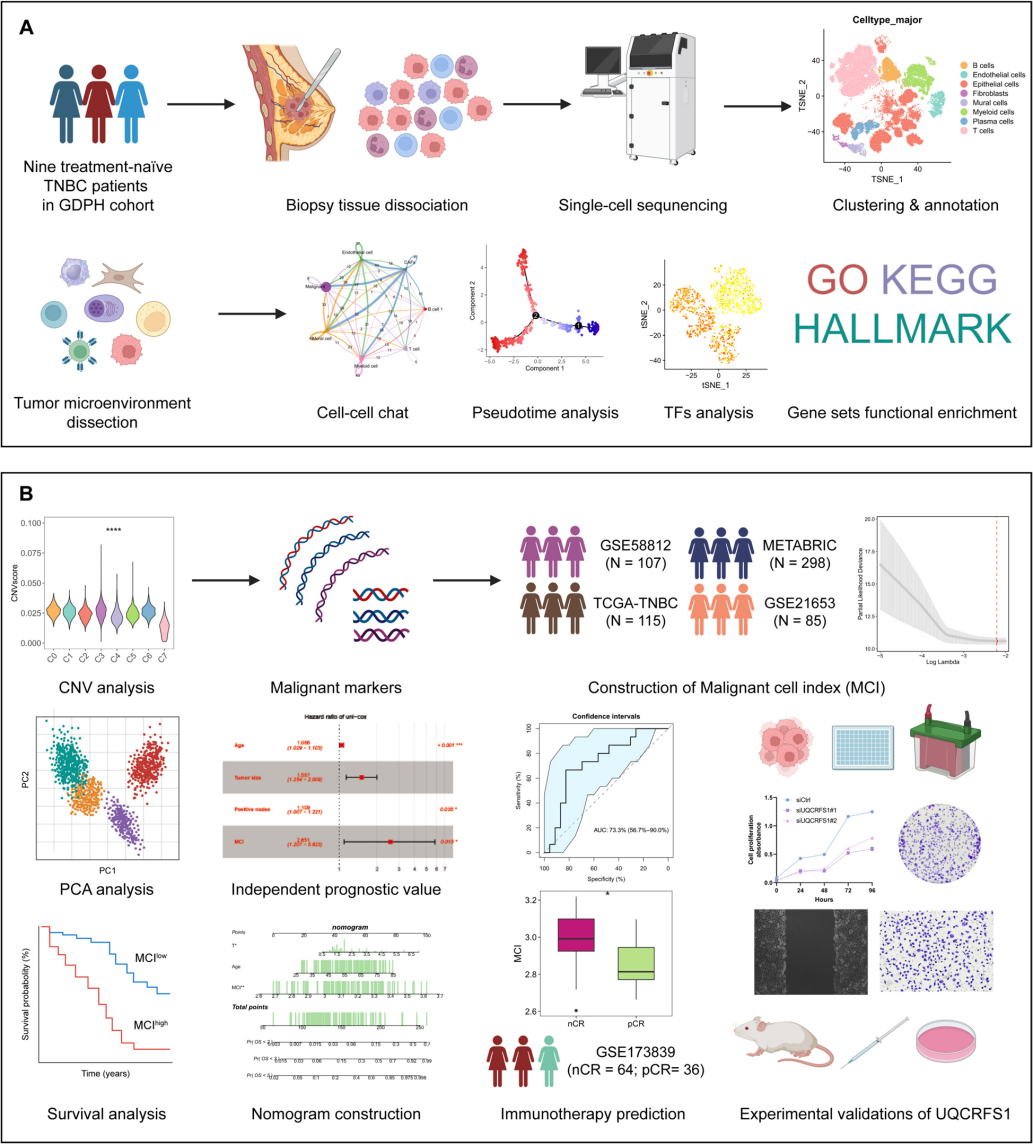
Overview of the study design and workflow based on the multi-omics analyses. **A** The biopsy tissues from nine treatment-naïve TNBC patients in Guangdong Provincial People’s Hospital (GDPH) were collected and subsequently dissociated for single-cell RNA sequencing (scRNA-sequencing). Additionally, the dissection of heterogenous tumor microenvironment (TME) was finalized by clustering, subclusters annotation, cell–cell chatting analysis, pseudotime analysis, TFs expression pattern and functional enrichment assays. **B** The malignant cell type was confirmed by CNV analysis and the Malignant Cell Index (MCI) was constructed by the expression of five key genes and was validated in four internal datasets using a series of algorithm analyses. Ultimately, the biological effect of UQCRFS1 was investigated through in vitro and in vivo experiments

The schematic representation of the study design and workflow was presented in Fig. 1A, B. The thorough analyses were conducted on the self-tested single-cell RNA sequencing dataset which contained nine TNBC treatment-naïve patients from Guangdong Provincial People's Hospital, and clarified eight major cell types. The interrelationships among the various major cell types were investigated by the CellChat algorithm and subsequently the subclusters were deciphered in every major cell type, which displayed diverse characteristics. Particularly, IL32^high^ Treg was corroborated to imparted a vital effect on tumor evasion and correlate with the worse prognosis of TNBC patients. Besides, a malignant epithelial cluster was unraveled using the copy number variations analysis. Additionally, we performed the LASSO-Cox regression algorithm to construct a malignant cell index (MCI) based on five genes (BGN, SDC1, IMPDH2, SPINT1, and UQCRFS1) from the differentially expressed genes in the malignant tumor cells and validated its clinical significance in four independent TNBC cohorts (GSE58812, TCGA-TNBC, METABRIC, and GSE21653), unveiling that the TNBC patients with a lower MCI had a favorable prognosis. Ultimately, a series of in vitro and vivo experiments were executed and unraveled that knockdown of iron-sulfur Rieske protein (UQCRFS1), which was a significant gene in MCI, curbed the proliferative and migratory characteristics of TNBC cells. The present study sheds more enlightenments on comprehending the heterogeneity within TNBCs, thereby facilitating the elucidation of TNBCs biology and providing clinical recommendations for TNBC patients' prognosis.

## Materials and methods

### Clinical samples attainment and silico data collection

The TNBC samples for scRNA sequencing were randomly selected from patients undergoing percutaneous biopsy and with treatment-naïve in the Department of Breast Cancer of Guangdong Provincial People’s Hospital, and the detailed clinical information of the candidate nine patients was shown in Table S1. All patients from Guangdong Provincial People’s Hospital provided informed consent for the utilization of specimens and the institutional review board of Guangdong Provincial People’s Hospital approved this study (KY-Z-2022-2545-02). And the single-cell library was constructed by Singleron Matrix single cell processing system as to the instructions of the GEXSCOPE Single Cell RNA Library Kits (Singleron Biotechnologies, Nanjing, China). Additionally, the raw scRNA sequencing data have been deposited in the Genome Sequence Archive in National Genomics Data Center, China National Center for Bioinformation/Beijing Institute of Genomics, Chinese Academy of Sciences (GSA: HRA008417) that are publicly accessible at https://ngdc.cncb.ac.cn/gsa.

The bulk RNA-seq transcriptome datasets of TNBC patients (GSE96058, GSE58812, TCGA-TNBC, METABRIC, GSE21653, GSE173839, and GSE76250) with the corresponding clinical data were collected from the Gene Expression Omnibus (GEO) database and the previous studies [7, 23–27]. “AnnoProbe” R package was applied to map the probes in transcriptomes. Besides, “limma” R package was utilized to cipher the median values of various probes in the same gene [28]. The TNBC proteomics dataset was retrieved from a previous study [29]. For spatial transcriptome (ST) data, a TNBC patient was acquired from Zenodo data repository (DOI: 10.5281/zenodo.4739739 ).

### scRNA‑seq and ST data preprocessing

“Seurat V4” R package were exploited to preprocess scRNA-seq and ST data [30]. All functions were implemented under the circumstance of their default parameters, unless particularly specified otherwise. Subsequently, it was excluded that a cell with the total gene count was less than 300 genes and a gene count was less than 3 cells per gene. And the count data of UMI was subjected to log-transformation for normalization. As a result, 2000 highly variable genes (HGVs) on the top were finally screen out. Besides, the matrix was scaled by segregating the centered expression by the standard deviation. The “harmony” R package was applied to mitigate batch effects among each patient [31]. The usage of the top 30 principal components got involved in the process, in conjunction with HGVs. The major cell clusters in the scRNA-sequencing were subsequently characterized utilizing the “FindClusters” function and displayed through the t-distributed stochastic neighbor embedding (tSNE) or the uniform manifold approximation and projection (UMAP) algorithm. Additionally, the annotation of clusters was finalized using classical markers. And the differentially expressed genes (DEGs) were evaluated by employing the “FindAllMarkers” function. For sub-clustering analysis, the same methodology was utilized, encompassing the identification of variable genes, reduction of dimensionality, and subsequent clustering.

For ST data, cell annotations were obtained from the published study and cells annotated with “artefact” had been filtered. Cell–cell chatting analysis between immune/stromal cells and epithelial cells was executed by “CellChat” R package [32]. The “inferCNV” R package was carried out to estimate CNV value for each region [17]. The CNV score for every single cell was determined by calculating an average value of the CNV region. The “Monocle2” package was utilized to conduct single cell trajectories [33]. TFs in Animal TFDB 2.0 were obtained and TF expression pattern analysis was carried out by “SCENIC” R package [34].

### Functional pathway enrichment analysis in different gene sets

The functional pathway enrichment analysis was finalized by “GSVA” and “clusterProfiler” R packages on the basis of a series of gene sets [35, 36], including HALLMARK (“h.all.v2023.1.Hs.symbols.gmt”), KEGG (“c2.cp.kegg.v2023.1.Hs.symbols.gmt”), and GO-BP (“c5.go.v2023.1.Hs.symbols.gmt”) signatures retrieved from MsigDB database. ssGSEA algorithm was implemented to evaluate the enrichment degree of the signatures of HALLMARK in each cell. Metabolic activity was assessed by “scMetabolism” algorithm [37].

### Construction of the MCI with malignant marker genes

Initially, the selection of malignant marker genes associated with survival was completed by dint of the univariate Cox regression model in GSE58812 and TCGA-TNBC datasets. And a cutoff of *p* value was set to 0.1 to avoid omissions. 227 of 1280 genes in GSE58812 and 117 of 1280 genes in TCGA-TNBC were screened out. Subsequently, we selected the common 28 genes of two cohorts and performed the logistical least absolute shrinkage and selector operation (LASSO)-Cox regression with the selection of “lambda. min” [38]. Ultimately, the model generated the MCI for every TNBC patient utilizing the following formula:

$$\text{MCI}= {\sum }_{i=1}^{5}\beta i*Ei$$

(βi notifies the coefficient index, and Ei demonstrates the gene expression level).

In order to enhance the intuitiveness of the plots, a linear transformation was employed to adjust the MCI. This involved subtracting the minimum value from the calculated MCI and dividing it by the maximum value, thereby mapping the resulting exponentials within the range of 0 to 1 [39].

According to the median MCI, TNBC patients were segregated into MCI^low^ and MCI^high^ subgroups within each cohort. The principal component analysis (PCA) was carried out with the utilization of “stats” R package. Additionally, the prognostic disparity between the two groups was discriminated by “survival” and “survminer” R packages.

### Independent prognostic value of the MCI

The clinicopathological data (such as age, tumor size, and positive nodes) of TNBC patients in the GSE58812 dataset were gathered and subsequently subjected to analysis alongside the MCI using the univariate and multivariable Cox regression methods.

### Development of the clinically predicted nomogram

The clinical nomogram was established using multivariable Cox regression and stepwise regression analyses, and the plot of nomogram was depicted by the “regplot” R package. The efficacy was figured out by the calibration plots and decision curve analysis (DCA) with the assist of the R packages “caret” and “rmda”. The R package “timeROC” was exploited to elicit the time-dependent receiver operating characteristic (ROC) curve [40].

### Potential exploration for TME landscape estimate based on the expression of UQCRFS1

To explore the correlation between the expression level of UQCRFS1 and TME heterogeneity, ESTIMATE algorithm [41] was utilized to evaluate the infiltration of immune cells (Immune score) and stromal cells (Stromal score) for further calculating tumor purity and analyzing TME between TNBC patients of METABRIC dataset with high- and low-expression of UQCRFS1. Besides, CIBERSORT deconvolution algorithm [42] was implemented to predict the abundance of 22 TME infiltrating cell types in the METABRIC-TNBC cohort.

### Cell lines and culture conditions

The normal mammary epithelial cell line MCF-10A and several TNBC cell lines including MDA-MB-468, MDA-MB-231, and BT549 were obtained from the American Type Culture Collection (ATCC). Moreover, all cell lines were cultured and maintained in accordance with the standard protocols, at a temperature of 37 °C and a relative humidity of 99%, without the addition of antibiotics. Additionally, two small interfering RNAs (siRNAs) and one short hairpin RNA (shRNA) against UQCRFS1 were biosynthesized through GenePharma corporation (Shanghai, China) and transfected through Lipofectamine 3000 (Invitrogen, CA, USA) according to the manufacturer’s protocol, and the detailed sequences of the siRNAs and shRNA included in this study are displayed in Table S2.

### RNA acquirement and qRT-PCR experiment

Total RNA from cells was acquired utilizing the RNA-Quick Purification Kit (Yishan Biotechnology Co., Shanghai, China). And the subsequent qRT-PCR experiment to assess mRNA expression by means of the SYBR Green method (Takara Bio Inc., Shiga, Japan) on a Bio-Rad CFX96, the primer sequences of which were provided in Table S3. Of note, the relative RNA expression was normalized against β-actin RNA and by dint of the 2^−ΔΔCt^ method.

### Western blotting analysis

Protein extracts from cells were collected by means of RIPA lysis buffer (Beyotime, Shanghai, China). Moreover, total proteins were applied to SDS-PAGE and subsequently transferred to PVDF membrane (Millipore, MA, USA). In addition, antibodies against UQCRFS1 and β-actin (CST, MA, USA) were utilized to incubate the membrane at 4 °C overnight and the following secondary antibody (CST, MA, USA) was similarly used at room temperature for 1 h. Ultimately, the blots were visualized by means of Immobilon Western Chemiluminescent HRP Substrate (Beyotime, Shanghai, China).

### Cell counting kit-8 (CCK-8) and colony formation assays

For CCK-8 assays, a total of 1500 TNBC cells per well were incubated in 96-well plates. Successively, the prepared CCK-8 solution (Beyotime, Shanghai, China) was added into each experimental well and continued to incubating for 2 h for assessing cell proliferative capacity. Finally, the measurement of absorbance at OD450 was finalized from days 0 to 4.

For colony formation assays, TNBC cells were incubated in 6-well plates at a density of 1,000 cells per well. The formative colonies were fixed utilizing methanol and stained with 0.1% crystal violet solution after 14 days incubation (Beyotime, Shanghai, China).

### Transwell assay

The transwell assay for assessing the migration of cells was conducted in accordance with the thorough process illustrated in the previous study [43]. In detail, a total of 50,000 cells were digested, resuspended and introduced into the upper chambers, which was treated without the addition of fetal bovine serum (FBS), and the lower cross-pore compartment was conversely treated with a solution with 20% FBS. After a period of 22-h culture, the cells in the transwell chambers were fixed with methanol and stained with 0.1% crystal violet (Beyotime, Shanghai, China) and subsequently imaged and counted to figure out the migratory features of TNBC cells.

### Animal experiments

The BALB/c female nude mice aged around three to four weeks were offered by Zhuhai BesTest Bio-Tech Co,. Ltd. (Zhuhai, China) and were further housed at the Center of Experimental Animals of Guangdong Provincial People's Hospital under the controlled conditions. For xenograft tumor models, the nude mice were subcutaneously injected with 1 × 10^6^ MDA-MB-231 or BT549 cells suspended in PBS with stable UQCRFS1 knockdown or the corresponding control cells, and were sacrificed after the observation and tumor volume measurement of 4 weeks. The Animal Care and Use Committee of Guangdong Provincial People’s Hospital granted approval for all procedures (KY-N-2022-054-02).

### Statistical analysis

The silico and statistical analyses in the present research were implemented by dint of R software (version 4.3.0) and GraphPad Prism software (version 8.0). Wilcoxon rank-sum test was exploited to compare the discrepancy between two groups in the present study. One-way ANOVA analysis was utilized in the comparison of three groups. The comparison of each Kaplan–Meier (K-M) curves in this study were performed by the log-rank test. And *p* < 0.05 was considered statistically significant.

## Results

### Single-cell atlas encompassing the cellular composition of TNBC patients

**Fig. 2:**
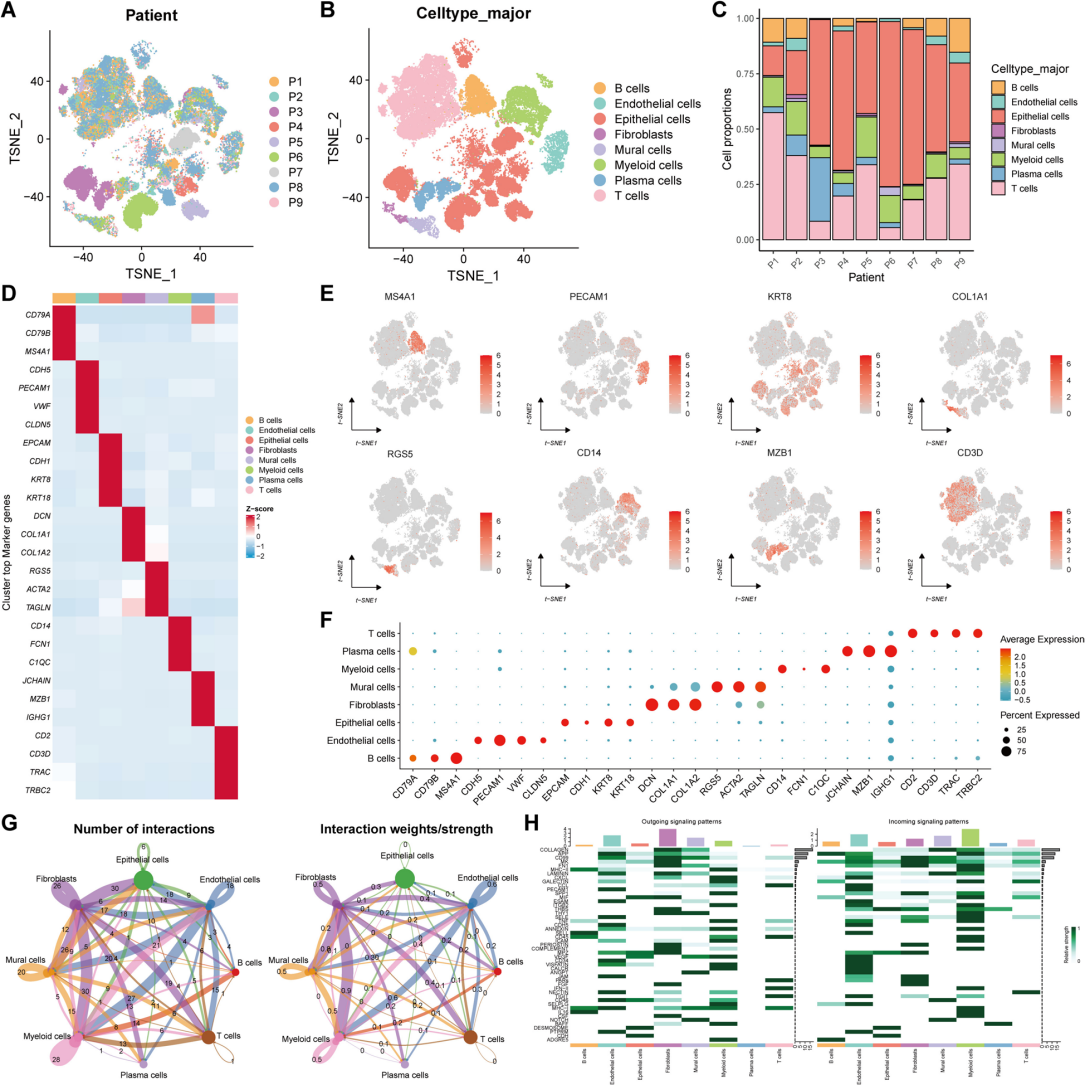
Determination of the major cellular compositions of TME in the single-cell atlas of TNBC. **A**, **B** tSNE plots revealing the patient labels (**A**) and the major cell types (**B**). **C** The proportional representation of eight major cell types across nine patients. **D**–**F** The top marker genes of every single major cell type in heatmap (**D**), tSNE plots (**E**), and bubble plot (**F**). **G** The cellular interactions among these major cell types in the quantitative (left) and intensity (right) levels. **H** The frequency of the top interaction genes of these major cell types in the outgoing (left) and incoming (right) signaling patterns

To dissect the heterogenous immune microenvironment of treatment-naïve TNBC, the biopsy tumors of nine patients were collected for single-cell RNA sequencing (Fig. 1A, B and Tab. S1). After eliminating the cells with low quality, a total of 92,304 cells were ultimately obtained, which were subsequently classified into eight major cell types, namely B cells, endothelial cells, epithelial cells, fibroblasts, mural cells, myeloid cells, plasma cells, and T cells. UMAP visualizations based on the label of patients or the major cell types were exhibited in Fig. 2A, B, and C disclosed that the proportional representation of eight major cell types across nine patients. Additionally, these major cell types were defined by the classic lineage markers of B cells (CD79A, CD79B, and MS4A1), endothelial cells (CDH5, PECAM1, VWF, and CLDN5), epithelial cells (EPCAM, CDH1, KRT8, and KRT18), fibroblasts (DCN, COL1A1, and COL1A2), mural cells (RGS5, ACTA2, and TAGLN), myeloid cells (CD14, FCN1, and C1QC), plasma cells (JCHAIN, MZB1, and IGHG1), and T cells (CD2, CD3D, TRAC, and TRBC2) (Fig. 2D–F). Besides, CellChat analysis was performed to identify a wide range of the varied and unique communications among these cell types of TME, revealing fibroblasts and myeloid cells obviously gained the highest interactions in the quantitative and intensity levels (Fig. 2G). Of note, fibroblasts displayed the highest frequency of the outgoing signal patterns while myeloid cells exhibited the utmost frequency of the incoming signal patterns, indicating their vital roles in TNBC microenvironment (Fig. 2H). To summarize, the heterogenous TME of TNBC was based on the diverse cell types and their mutually strong interactions.

### Characterization of myeloid cells in TNBC

**Fig. 3:**
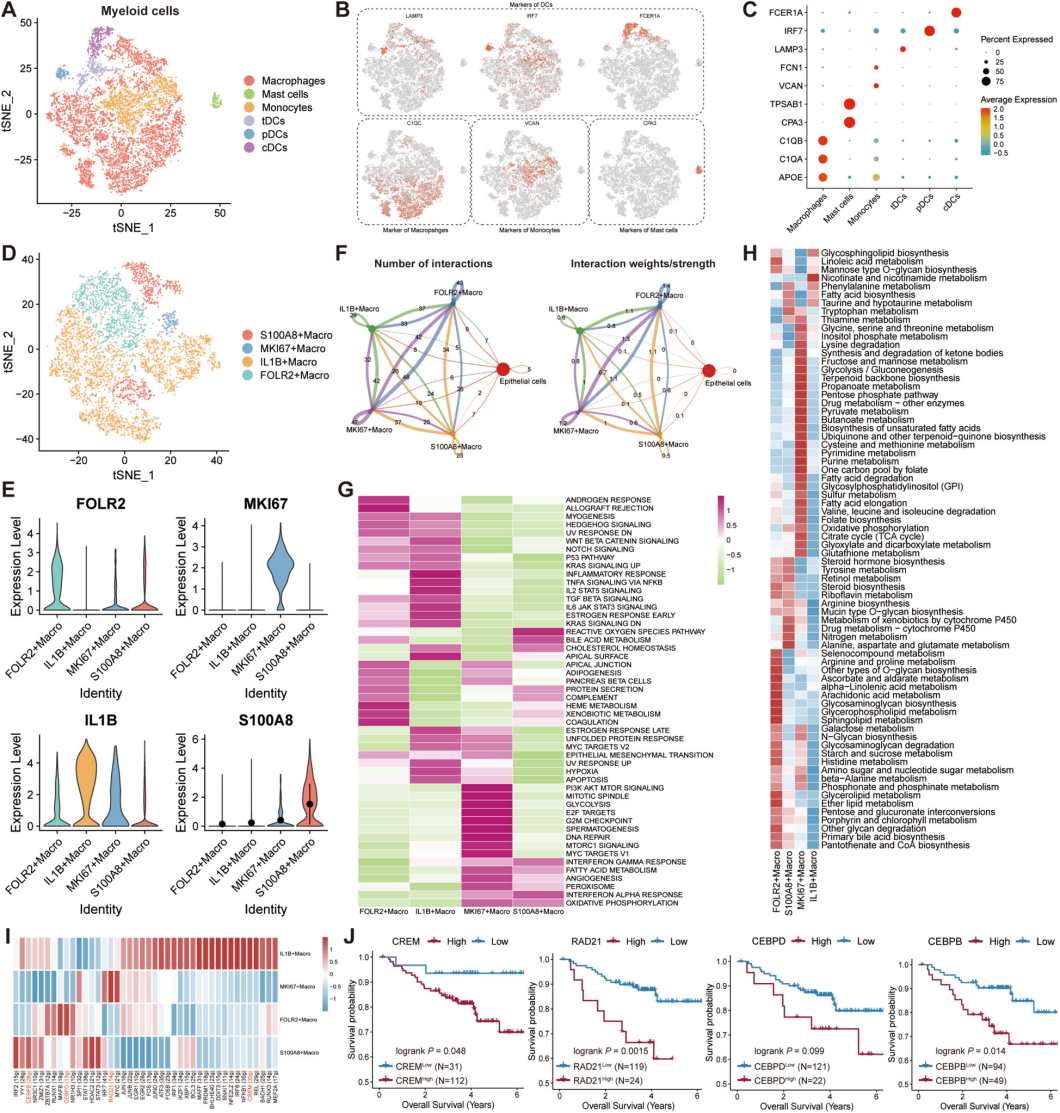
Clarification of myeloid cell subclusters in TNBC. **A** tSNE plot of the subclusters of myeloid cells. **B**, **C** The top marker genes of each subcluster of myeloid cells in tSNE plots (**B**) and bubble plot (**C**). **D** The detail subclusters of macrophages in tSNE plot. **E** The top marker genes of macrophage subclusters in violin plots. **F** The cellular interactions among macrophage subclusters in the quantitative (left) and intensity (right) levels. **G** The functional pathways enrichment analysis using HALLMARK gene sets in macrophage subclusters. **H** The metabolic pathway enrichment analysis through scMetabolism algorithm in macrophage subclusters. **I** TFs expression pattern of macrophage subclusters. **J** Survival analyses of the specific TF marking every single macrophage subcluster in GSE96058 dataset

Based on the tSNE algorithm, the myeloid cells were reclustered into six subclusters, namely macrophages, mast cells, monocytes, tolerogenic dendritic cells (tDCs), plasmacytoid DCs (pDCs), and conventional DCs (cDCs) (Fig. 3A), and the markers of the subclusters were shown in Fig. 3B, C. We then focused on the macrophages and corroborated the four subclusters, including S100A8^+^ Macro, IL1B^+^ Macro, FOLR2^+^ Macro, and MKI67^+^ Macro (Fig. 3D). The expression levels of markers in every subcluster were demonstrated in violin plots, which confirmed the accuracy of clustering (Fig. 3E). Moreover, Cellchat analysis uncovered that each subcluster of microphages had wide communications among themselves (Fig. 3F). Subsequently, functional pathway enrichment analysis was executed to explore the potential relevant signaling pathways and biological functions of each subcluster using hallmark pathway sets (Fig. 3G). In detail, FLOR2^+^ Macro attained the enrichment of androgen response, allograft rejection; IL1B^+^ Macro obtained the enrichment of apoptosis, hypoxia, inflammatory response, etc.; MKI67^+^ Macro got hold of the enriched pathways of DNA repair, mitotic spindle, G2M checkpoint, etc.; S100A8^+^ Macro was primarily enriched in reactive oxygen species pathway, oxidative phosphorylation, etc. Afterwards, the differences of metabolic pathways among each subcluster of macrophages were analyzed, which indicated that FOLR2^+^ and MKI67^+^ Macro subclusters acquired the relatively enhanced activities of metabolic pathways while S100A8^+^ and IL1B^+^ Macro subclusters displayed the lower activities (Fig. 3H). Additionally, TF analysis among each subcluster was performed and it was noteworthy that TFs of CREM, PDRM1, REL, IRF8, etc. were significantly promoted in the IL1B^+^ Macro; RAD21 and MYC were primarily enhanced in the MKI67^+^ Macro; CEBPD and MAFB were considerably augmented in the FOLR2^+^ Macro; CEBPBM, YY1, IRF2, etc. were appreciably upregulated in the S100A8^+^ Macro (Fig. 3I). The Kaplan–Meier (K-M) analysis for the overall survival based on these specific TFs among the subcluster of macrophages in GSE96058 and MATABRIC-TNBC cohorts validated that these TFs served as the essentially detrimental prognosis-factors for TNBC patients (Fig. 3J and S1). In a nutshell, the distinct characteristics of myeloid cells, especially macrophages, were confirmed in TME of TNBC, including functional pathways and TF expression, which hinted a host of macrophages were correlated with the shorten prognosis of TNBC.

### Diversity of fibroblasts and B/plasma cells in TNBC

**Fig. 4:**
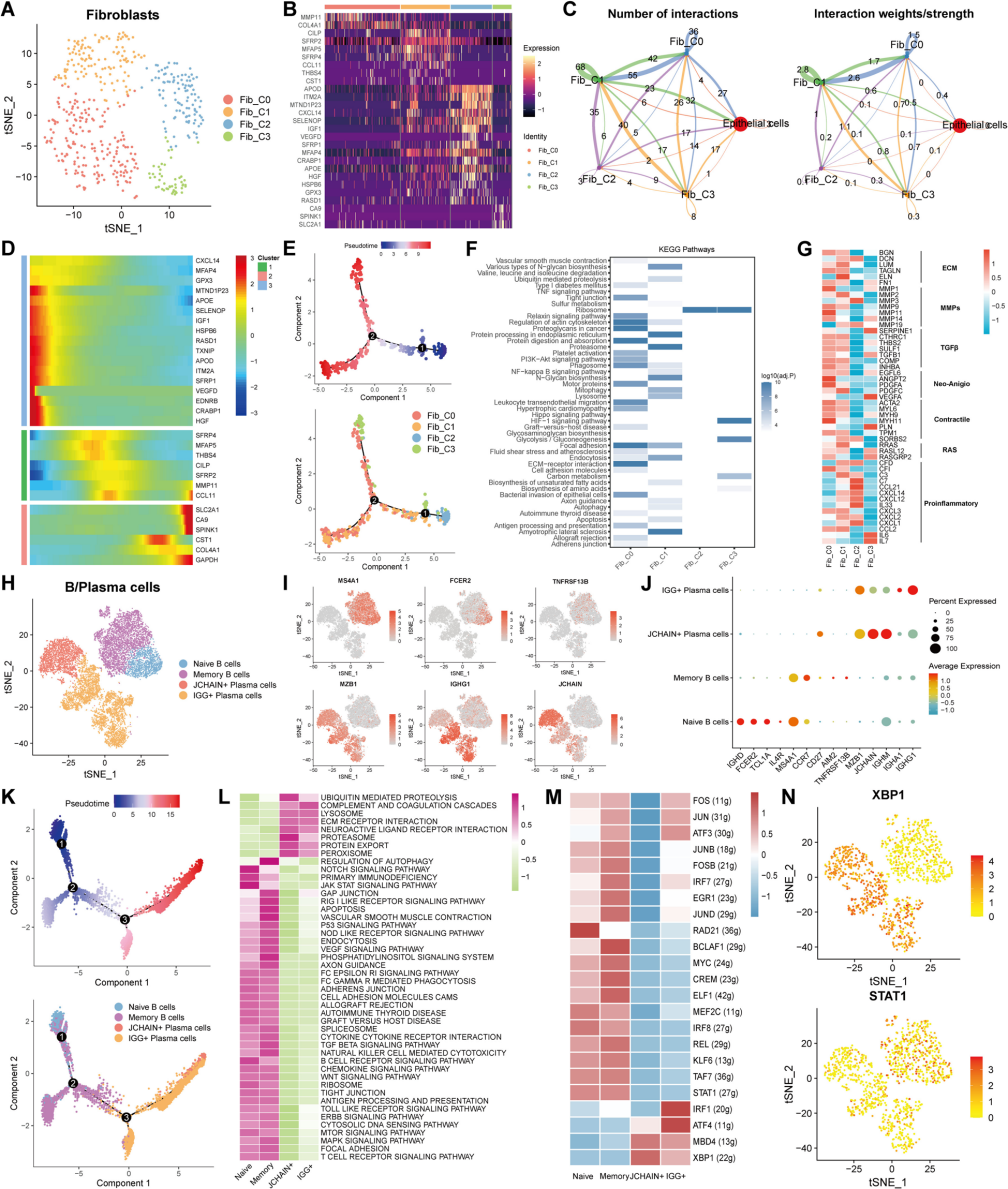
Characterization of fibroblasts and B/plasma cells in TNBC. **A** tSNE plot unveiling the subgroups of fibroblasts. **B** The top marker genes of fibroblast subclusters in heatmap. **C** The cellular interactions among fibroblast subclusters in the quantitative (left) and intensity (right) levels. **D**, **E** The top genes expression related to differentiation process (**D**) and the differentiation trajectory plots (**E**) of fibroblast subclusters through pseudotime analysis. **F** Functional pathways enrichment analysis in fibroblast subclusters using KEGG dataset. **G** The expression patterns of the genes from extracellular matrix (ECM), matrix metalloproteinases (MMPs), TGF-β, Neo- angiogenesis (Neo-Anigio), contractile, RAS, and proinflammatory processes in fibroblast subclusters. **H** The subclusters of B/plasma cells in tSNE plot. **I**, **J** The top marker genes of B/plasma cell subclusters in tSNE plots (**I**) and bubble plot (**J**). **K** The differentiation trajectory plots of B/plasma cell subclusters by pseudotime algorithm. **L** The functional pathways enrichment analysis in B/plasma cell subclusters based on HALLMARK database. **M** TFs expression pattern of B/plasma cell subclusters. **N** tSNE plots unraveling the expression of XBP1 and STAT1 in B/plasma cells

The accumulating evidence illustrated that fibroblasts of the primary or metastatic tumors exhibited the remarkable adaptability, plasticity, and resilience, actively contributing to the tumor progression by involving in the intricate interactions with various cell populations within the TME [44]. We reclustered the fibroblasts and clarified four clusters (Fib_C0—C3) based on the tSNE algorithm (Fig. 4A). Heatmap displayed the differentially expressed genes among these clusters (Fig. 4B). Cellchat analysis signified that Fib_C0 and Fib_C1 clusters possessed more communications compared with Fib_C2 and Fib_C3 clusters (Fig. 4C). Pseudotime analysis indicated the presence of a distinct differentiation trajectory from Fib_C2 to other clusters (Fig. 4D and E). In addition, functional pathways enrichment analysis was implemented based on the KEGG database, unraveling that Fib_C0 and Fib_C1 clusters enriched the numerous pathways, like focal adhesion, the regulatory process of actin cytoskeleton, phagosome, etc., while Fib_C2 and Fib_C3 clusters gained the relatively less pathways (Fig. 4F). Furthermore, some surface protein genes expression among four clusters were compared, which identified that Fib_C0 and Fib_C1 clusters were associated with multiple genes from extracellular matrix (ECM), matrix metalloproteinases (MMPs), TGF-β, Neo-Anigio, contractile, RAS, and proinflammatory while Fib_C2 was more likely to be correlated with proinflammatory (Fig. 4G).

Afterwards, we verified two B cells subclusters (naïve and memory B cells) and two subclusters of plasma cells (JCHAIN^+^ and IGG^+^ plasma cells) (Fig. 4H), and the markers of each subcluster were portrayed in Fig. 4I, J. Monocle analysis elucidated the existence of the differentiated process from B cells to plasma cells (Fig. 4K). Besides, pathway enrichment analysis enciphered that the naïve and memory B cells were endowed by the promoted activities of immune-regulated pathways like natural killer (NK) cell mediated cytotoxicity, T cell receptor signaling pathway, etc., whereas JCHAIN^+^ and IGG^+^ plasma cells were characterized in the reinforced activities of ubiquitin mediated proteolysis, complement, and coagulation cascades, etc. (Fig. 4L) Subsequently, TF analysis elaborated that plasma cell subclusters had the enhanced expression of XBP1 while the naïve and memory B cell subclusters possessed the elevated expression of STAT1 (Fig. 4M, N), which were both key modulators in the differentiated process of B cells. Collectively, the diversity of fibroblasts and B cells in TME of TNBC was illuminated in the aspects of the differentiation trajectory, functional pathways and TFs expression.

### T/NK cells are distinguished in TNBC

**Fig. 5:**
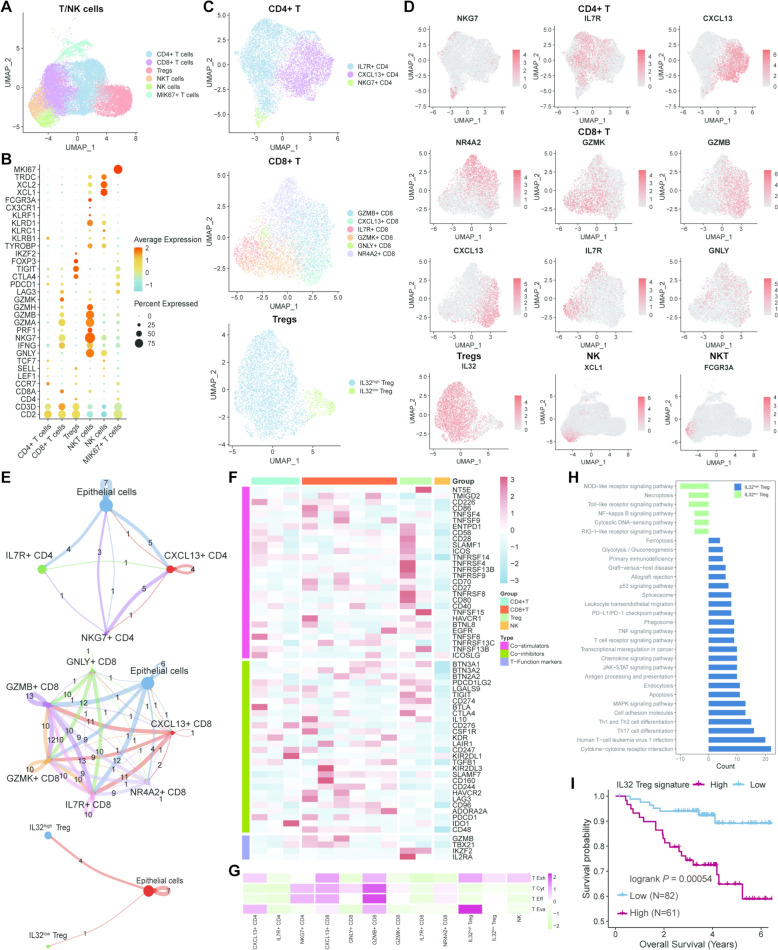
Verification of the heterogenous T/NK cells in TNBC. **A** UMAP plot indicating the heterogeneity of T/NK cells. **B** The top marker genes of T/NK cell subclusters in bubble plot. **C** The reclustering results of CD4^+^ T (top), CD8^+^ T (middle), and Tregs (bottom) cells in UMAP plots. **D** The top marker genes of T/NK cell subclusters in UMAP plots. **E** The cellular interactions among the subclusters of CD4^+^ T (top), CD8^+^ T (middle), and Tregs (bottom) as well as epithelial cells in the quantitative levels. **F** The expression patterns of genes related to the co-stimulation, co-inhibition, and certain function of T cells in T/NK cell subclusters. **G** The functional scores correlated to T exhaustion, T cytotoxic process, T effector and T evasion of the detailed subclusters of T/NK cells. **H** The differentially functional pathways related to the immune regulation in IL32^high^ and IL32^low^ Tregs. **I** Survival analysis based on the score of IL32^high^-Tregs-related signature of TNBC patients in GSE96058

T cells and NK cells are also regarded as the crucial components in the TME. We screened out T/NK cells and renamed the idents based on tSNE algorithm, identifying CD4^+^ T cells, CD8^+^ T cells, regulatory T cells (Tregs), MKI67^+^ T cells, NK cells, and NKT cells (Fig. 5A), and the markers of each cell type were presented in Fig. 5B. Then, the corresponding subclusters of CD4^+^ T cells, CD8^+^ T cells, and Tregs were exhibited in Fig. 5C, D, including three CD4^+^ T cells subtypes (IL7R^+^, CXCL13^+^, and NKG7^+^ CD4^+^ T cells), six CD8^+^ T cells subtypes (GZMB^+^, CXCL13^+^, IL7R^+^, GZMK^+^, GNLY^+^, and NR4A2^+^ CD8^+^ T cells), and two Tregs subtypes (IL32^high^ and IL32^low^ Tregs). And successively, Cellchat analyses unearthed the communicational differences among these subtypes and epithelial cells (Fig. 5E), respectively implicating CXCL13^+^ CD4^+^ T, GZMB^+^ and IL7R^+^ CD8^+^ T, as well as IL32^high^ Treg acquired the apparently more interplay with epithelial cells. Furthermore, in order to evaluate the comprehensive impacts of each T/NK subtype, the numerous disparities were investigated in the expression levels of immune genes associated with co-stimulation, co-inhibition, and certain function-related markers (Fig. 5F). And the significant differences were also observed in T-cell-related functional signature scores across these distinct subtypes, encompassing the scores of the exhaustion, cytotoxicity, effector, and evasion of T cells (Fig. 5G), which unveiled that CXCL13^+^ CD8^+^ T cell obtained the top mark in T exhaustion; GZMK^+^ CD8^+^ T cell exerted the most effects to T cytotoxic and effective process; IL32^high^ Treg essentially functioned in the process of T evasion. Subsequently, the differentially functional pathways were compared between the IL32^high^ and IL32^low^ Tregs in Fig. 5H, which depicted that IL32^high^ Tregs enriched more immune deregulation related pathways than IL32^low^ Tregs, such as translational mis-regulation process, PD-L1/PD-1 checkpoint pathway and primary immunodeficiency. Ultimately, we calculated the score of IL32^high^-Tregs-related signature of TNBC patients in GSE96058 and validated that the more IL32^high^ Treg infiltration in TME was associated with the worsen prognosis of TNBC patients (Fig. 5I). In summary, there existed the tremendous heterogeneity in immune cells of TNBC microenvironment and particularly IL32^high^ Treg might take a crucial part in immune evasion, which predicted an impaired prognosis in TNBC patients.

### Discrimination of subgroups in the TNBC epithelial cluster

**Fig. 6:**
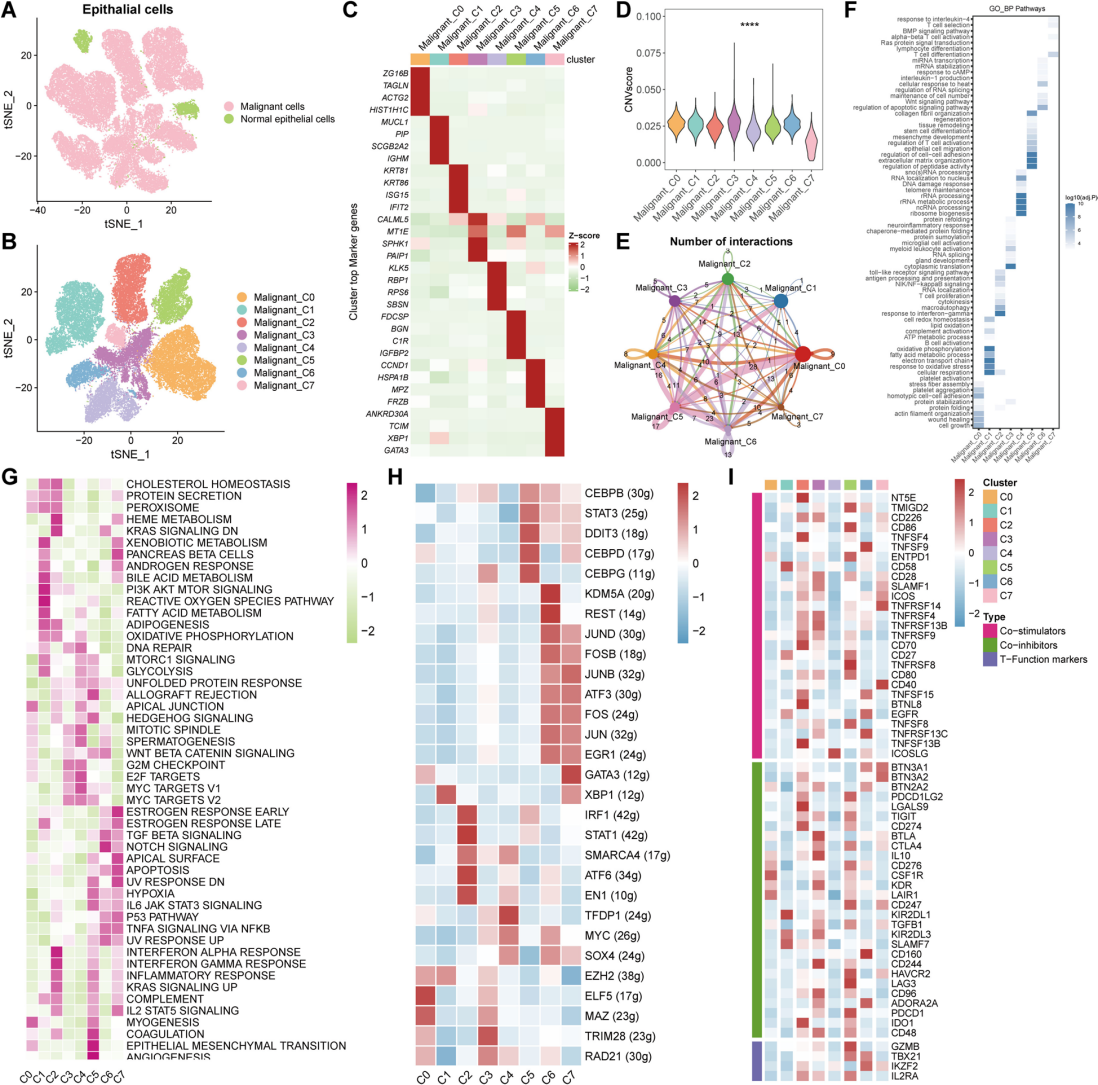
Discrimination of the normal and malignant epithelial cells in TNBC. **A** tSNE plot disclosing the normal and malignant cells in epithelial cells using “inferCNV” algorithm. **B** The reclustering result of malignant cells in tSNE plot. **C** The top marker genes of malignant cell subclusters in heatmap. **D** The CNV score of malignant cell subclusters. **E** The number of interactions among malignant cell subclusters. **F**, **G** The functional pathway enrichment of malignant cell subclusters using GO_BP (**F**) and HALLMARK (**G**) gene sets. **H** TFs expression pattern of malignant cell subclusters. **I** The expression patterns of genes related to the co-stimulation, co-inhibition, and certain function of T cells in malignant cell subclusters

In order to ascertain the heterogeneity of epithelial cells in TNBC, the normal and malignant epithelial cells were recognized by dint of the “inferCNV” algorithm, and a reclustering analysis was conducted in the malignant cells to identify eight subclusters (Malignant_C0–C7) (Fig. 6A, B). And the markers of each cluster were portrayed in Fig. 6C. Then, copy number variant (CNV) score was compared among the subclusters of the malignant cells, enlightening that Malignant_C3 displayed significantly reinforced levels of CNV score contrasting to other clusters (Fig. 6D). And successively, CellChat analysis delineated that Malignant_C5 possessed the utmost interactive numbers among these subclusters (Fig. 6E). Besides, the functional enrichment analysis using GO and hallmark databases implied that Malignant_C5 participated in the modulation of T cell activation, cell–cell adhesion and ECM organization as well as epithelial mesenchymal transition (EMT); Malignant_C2 primarily functioned at the response of interferon alpha and gamma; Malignant_C1 played a potential role in the regulation of metabolic pathways, consisting of fatty acid metabolism, glycolysis, oxidative phosphorylation, and reactive oxygen species pathway (Fig. 6F, G). Moreover, the different TF activities and the expression levels of immune genes associated with co-stimulation, co-inhibition, and the certain function-related markers in subclusters of the malignant cells were verified, which indicated that TFs of CCAAT enhancer binding proteins family and a host of immune regulatory genes were augmented in Malignant_C5 and the pivotal TFs related to interferon response, like STAT1 and IRF1, were substantially promoted in Malignant_C2 (Fig. 6H, I). Briefly, the distinct features of the subclusters within the malignant cells of TNBC probably imparted the indispensable effects to the development of TME by means of the diverse functional pathways.

### Construction and validation of MCI rooted on the malignant epithelial markers in TNBC

**Fig. 7:**
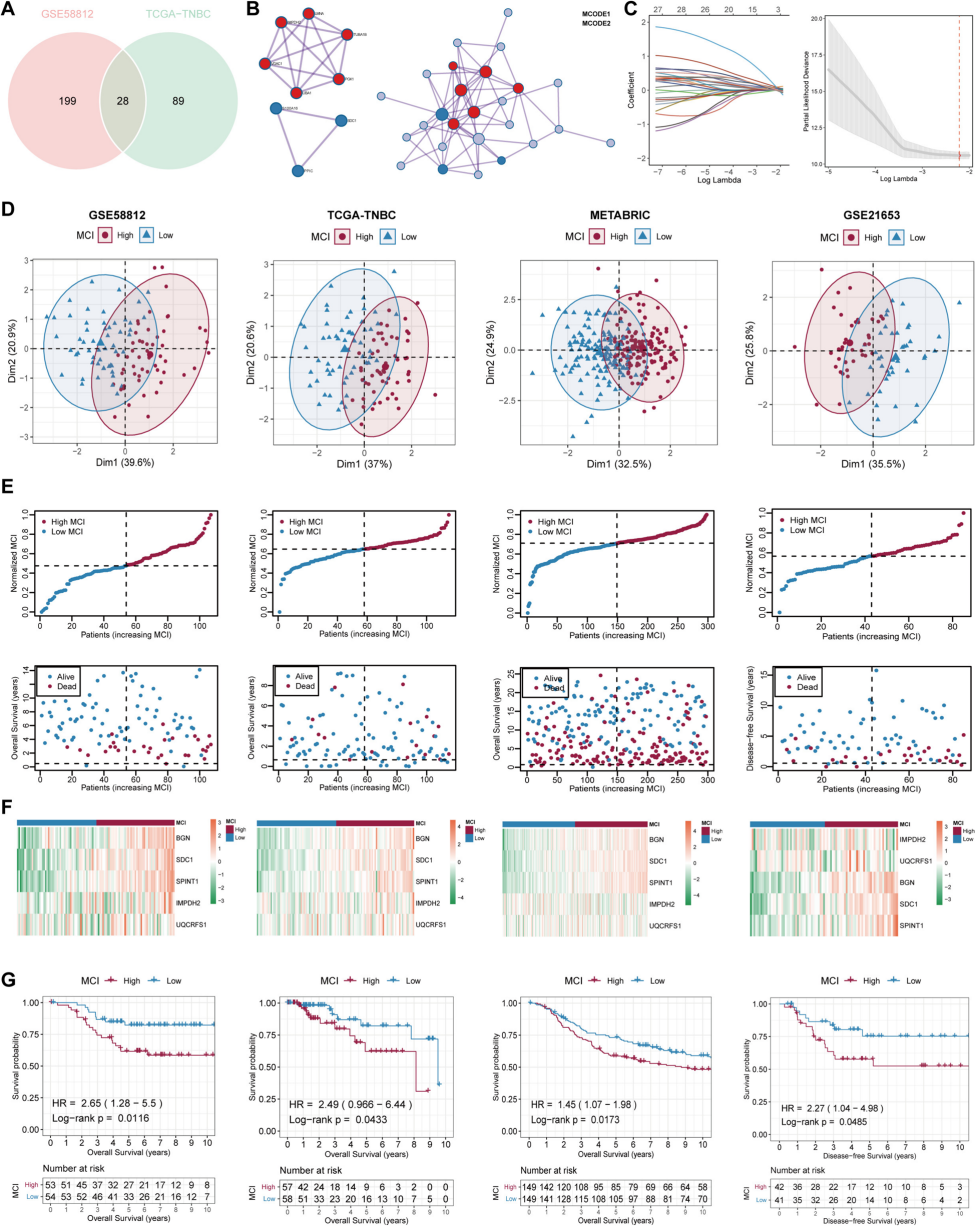
Construction and validation of Malignant Cell Index (MCI) model in TNBC patients. **A** Venn diagram depicting the clinically significant genes promoted in the malignant cells of scRNA-sequencing after performing univariate Cox regression in GSE58812 and TCGA-TNBC cohorts. **B** The interaction network of 28 overlapping genes. **C** The result of Lasso Cox regression analysis using these 28 genes in GSE58812 (left) and the partial likelihood deviance for the Lasso regression (right). **D** PCA analysis based on the MCI value of TNBC patients in the training and validation cohorts. **E** The adjusted MCI value (top) and the dead/alive status (bottom) of TNBC patients in the training and validation cohorts. **F** Heatmaps illustrating the expression patterns of MCI-included genes (BGN, SDC1, IMPDH2, SPINT1, and UQCRFS1) in MCI^high^ and MCI^low^ groups. **G** Survival analyses based on the comparison between patients with MCI^high^ and MCI^low^ values

Aiming to investigate the potential clinical utility of gene expression patterns in malignant epithelial cells which significantly promoted 1280 genes in scRNA-sequencing data, univariate Cox regression and LASSO algorithms were employed to gain the actual prognostic malignant genes in the bulk transcriptome data of TNBC patients. The training sets comprised of the GSE58812 and TCGA-TNBC datasets, whereas the validation sets consisted of the METABRIC and GSE21653 datasets. After implementing the univariate Cox regression and overlapping analysis, 28 common genes were screen out in the training sets (Fig. 7A, B) and ultimately five genes were selected to construct the malignant cell index (MCI) utilizing LASSO algorithms (Fig. 7C). The formula was MCI = (0.1068) * BGN + (0.0572) * SDC1 + (0.0065) * IMPDH2 + (0.1193) * SPINT1 + (0.0200) * UQCRFS1. Moreover, TNBC patients were segregated into MCI^high^ and MCI^low^ subgroups rooted on the median value of MCI in all datasets.

Next, PCA analysis was finalized in each cohort and disclosed that the distinguishments based on MCI in the training and validation cohorts were all satisfactory (Fig. 7D). Besides, MCI values were manually adjusted from 0 to 1 to make data straightforward and the proportion of dead patients probably was more in MCI^high^ group compared to MCI^low^ group (Fig. 7E). Furthermore, the expression pattern of five key genes in MCI were displayed between MCI^high^ and MCI^low^ groups to identify that these genes were all upregulated in MCI^high^ patients of TNBC (Fig. 7F). Additionally, K-M survival analyses for overall survival based on MCI values illuminated that there was a significantly reduced survival rate among TNBC patients with MCI^high^ levels in contrast to those with MCI^low^ values in each cohort (Fig. 7G). In general, the internal trainings and external validations suggested that MCI had a promising potential in the monitoring of survival outcomes of TNBC patients.

### Independent prognostic value of the MCI and establishment of the nomogram

**Fig. 8:**
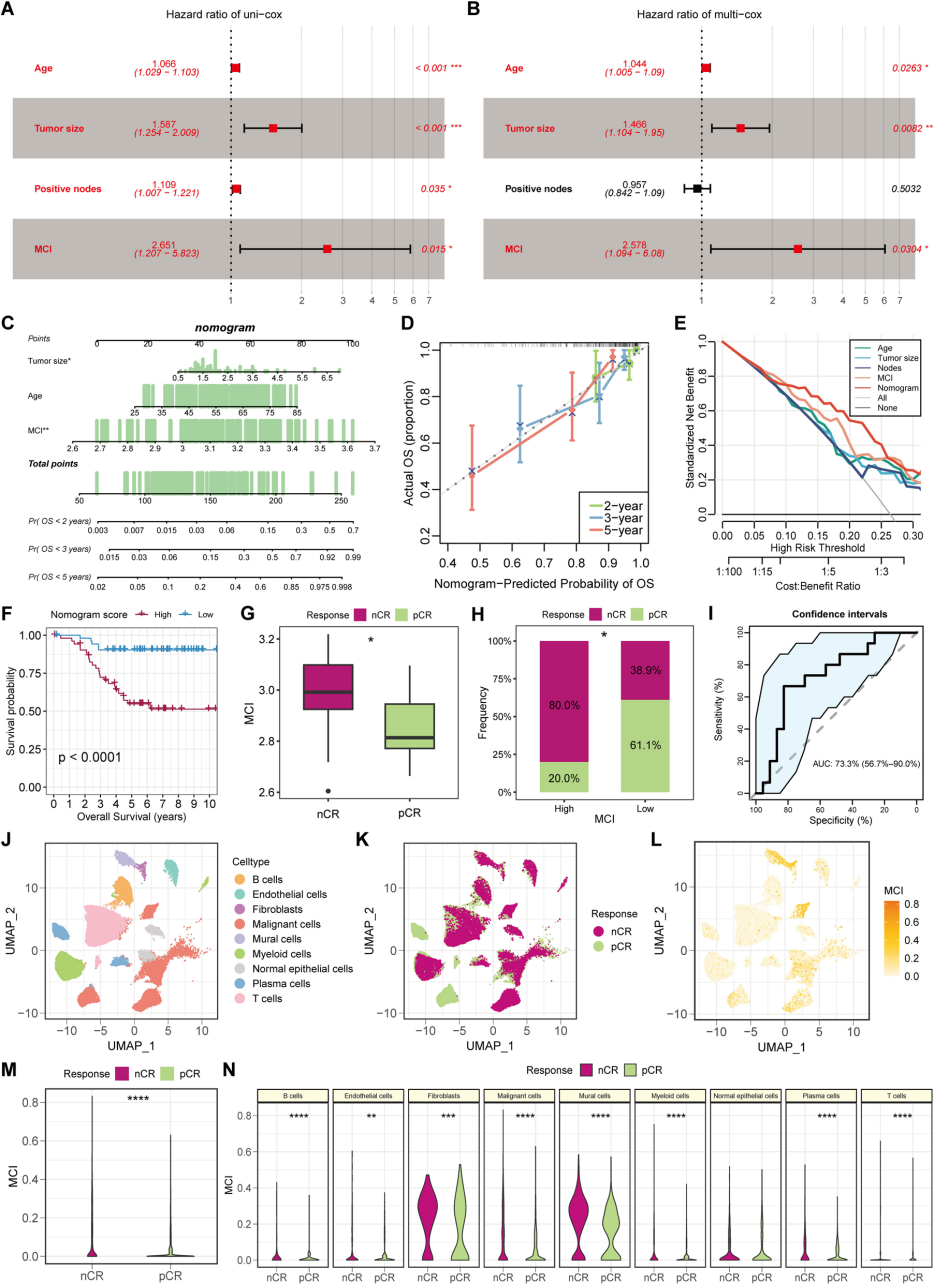
Establishment of a clinical nomogram including MCI and the significance of MCI in predicting immunotherapy resistance. **A**, **B** The results of univariate Cox (**A**) and multivariate Cox (**B**) regression utilizing age, tumor size, positive nodes and MCI of TNBC patients in GSE58812. **C** Construction of a prognostic nomogram consisting of MCI to predict 2-, 3-, and 5-year OS in TNBC patients. **D** Calibration curve to evaluate the congruity between the projected and observed rates of survival. **E** Decision curve analysis (DCA) to assess the clinical decision-making benefits of the nomogram. **F** Survival analysis based on the patients with high- and low- nomogram score. **G** The comparison of MCI of patients between nCR and pCR groups in GSE173839. **H** The proportion of patients with nCR or pCR status in MCI^high^ and MCI^low^ groups. **I** ROC analysis to evaluate the accuracy of MCI applied in immunotherapy response prediction. **J**–**L** UMAP plots displaying the major cell types (**J**), nCR/pCR status (**K**), and MCI values (**L**) of seven TNBC patients undergoing neoadjuvant ICB therapy in GDPH cohort. **M**, **N** The MCI values of total cell types (**M**) and the different major cell types (**N**) between nCR and pCR groups of GDPH cohort. * means *p* < 0.05, ** means *p* < 0.01, *** means *p* < 0.001, **** means *p* < 0.0001

To explore the independent prognostic essentiality of MCI, univariate and multivariable Cox regression analyses were implemented on the basis of several clinical characteristics including age, tumor size, positive nodes, and MCI. Forest plots illustrated that MCI was an independent prognostic indicator in both univariate and multivariable Cox regression, enabling to predict the inferior survival of TNBC patients in GSE58812 cohort (Fig. 8A, B). Furthermore, multivariable Cox and stepwise regression analyses were carried out to construct a nomogram model within the GSE58812 cohort, which was based on the age, tumor size, and MCI and enabled to estimate the likelihood of 2-, 3-, and 5-year OS (Fig. 8C). The calibration curves were utilized to evaluate the congruity between the projected and observed rates of survival using the nomogram model, which demonstrated the satisfactory predictive discrimination in TNBC patients (Fig. 8D). Besides, decision curve analysis (DCA) was applied to validate a spectrum of threshold probabilities for the nomogram, discerning that the nomogram possessed the superior performance compared to all other predictors employed in this investigation (Fig. 8E). Moreover, we calculated the nomogram score of each sample and ciphered that TNBC patients with the low nomogram score had the prolonged prognosis than those with the high score, which was accordance with the findings above (Fig. 8F). Concisely, MCI indeed served as a clinically crucial indicator for the worsen prognosis of TNBC patients and the constructed nomogram consisting of MCI also gained the perfect stability and practicality in predicting the impaired prognosis of TNBC patients.

### MCI enables to predict the immunotherapy response of TNBC patients

TNBC is a highly aggressive subtype of BC featured by a scarcity of effective treatment modalities, and the appearance of immune checkpoint blockade (ICB) therapy exhibits the tremendous potential in TNBC treatment [45]. To explore whether MCI is linked to the prediction of immunotherapy response, the transcriptome data of GSE173839 which contained TNBC patients treated with ICB (durvalumab with olaparib) and paclitaxel were collected. And then, the MCI of each TNBC patient was calculated in GSE173839, prompting that the TNBC patients without pathologic complete response (nCR) to ICB attained the higher MCI values contrasting to those with pathologic complete response (pCR) (Fig. 8G). Subsequently, TNBC patients were similarly segregated into MCI^high^ and MCI^low^ subgroups based on the median value of MCI, exploring that the proportion of patients with nCR in the MCI^high^ subgroup was significantly larger than that in the MCI^low^ group (Fig. 8H). Additionally, ROC analysis was executed and illustrated that the area under the ROC curve (AUC) value was 0.733, which proved the accuracy of MCI applied in immunotherapy response prediction (Fig. 8I).

Furthermore, the findings above were validated in our scRNA-seq cohort, which contained seven patients treated with neoadjuvant ICB therapy and chemotherapy after definitely diagnosing as TNBC tumor (Fig. 8J and Table S1). Of note, four of them achieved pCR status while others remained nCR, which were presented in the UMAP plot (Fig. 8K). In addition, the distribution of MCI in the total major cell types were portrayed in Fig. 8L, and the comparison of MCI between nCR and pCR groups hinted that TNBC patients with nCR to immunotherapy obtained the higher accumulative MCI than those with pCR status based on the scRNA data, in line with the forementioned results (Fig. 8M). In detail, MCI values were compared between the different response outcome in every single major cell type, unraveling that MCI values were significantly promoted in the nCR group within the most cell types including malignant cells, except for the normal epithelial cells (Fig. 8N). Collectively, these findings indicated that MCI could predict the cacoethic immunotherapy response of TNBC patients.

### UQCRFS1 is a potential biomarker in TNBC

**Fig. 9:**
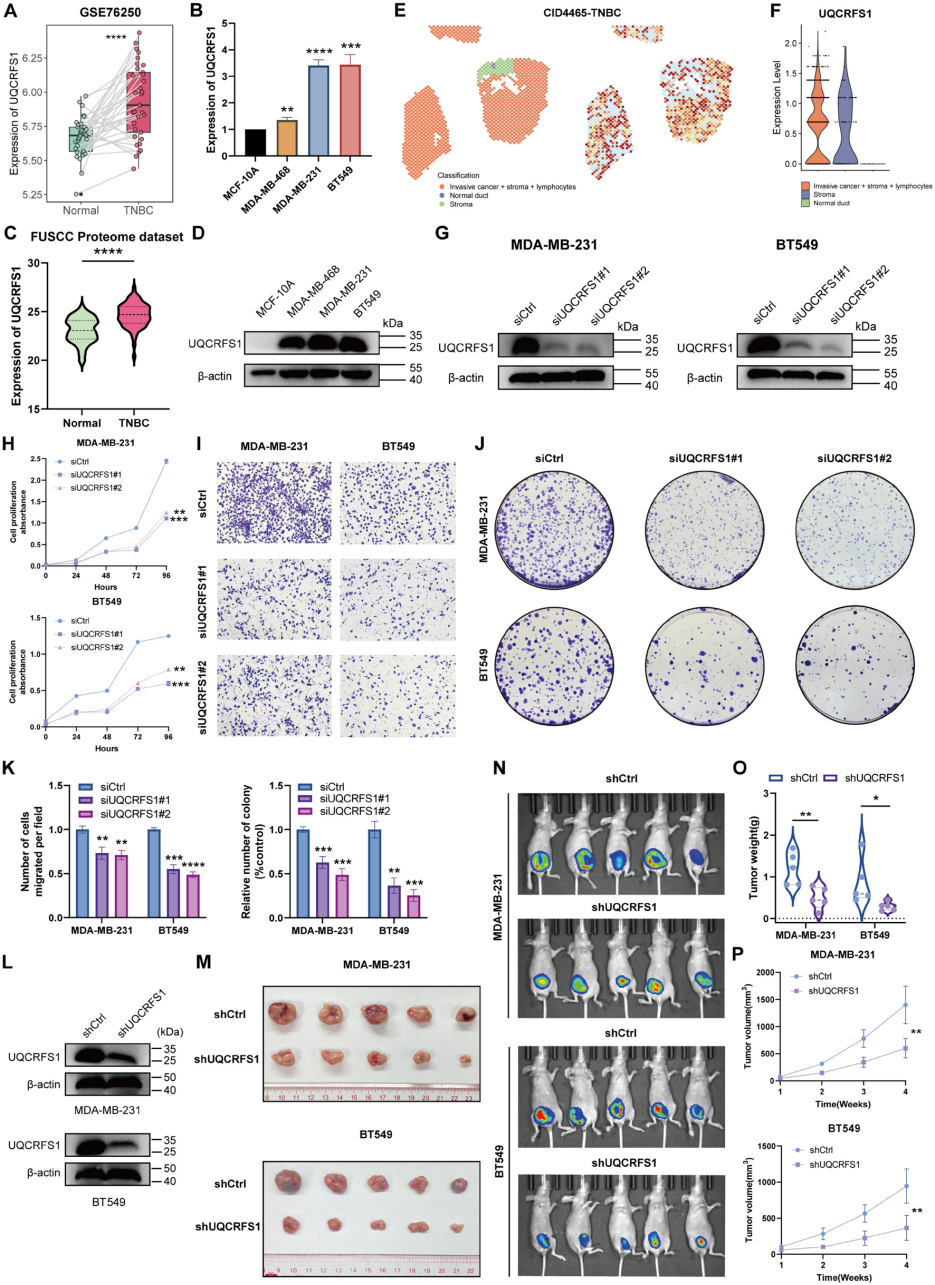
UQCRFS1 is significantly enhanced in TNBC samples and potentiates tumor progression. **A** The expression level of UQCRFS1 in the paired non-tumoral and TNBC samples of GSE76250 dataset. **B** qRT-PCR analysis validating the mRNA expression of UQCRFS1 in MCF-10A and TNBC cell lines. **C** The protein expression of UQCRFS1 in the normal and TNBC samples of FUSCC proteome dataset. **D** Western blotting analysis validating the protein level of UQCRFS1 in MCF-10A and TNBC cell lines. **E** The classification of different spatial areas (left) and the expression of UQCRFS1 (right) in spatial transcriptome of a TNBC patient. **F** The statistical level of UQCRFS1 expression in (**E**). **G** Validation of two siRNAs targeting UQCRFS1 in MDA-MB-231 and BT549 cells. **H** The result of CCK-8 assays under the situation of UQCRFS1 knockdown. **I**, **J** The representative pictures of transwell (**I**) and colony formation (**J**) assays. **K** The statistical results of transwell (left) and colony formation (right) assays. **L** Validation of the shRNA targeting UQCRFS1 in TNBC cells. **M** The representative images of xenograft tumor morphology using MDA-MB-231 (top) and BT549 (bottom) cells. **N** The representative images of optical luciferase imaging assays in vivo. **O**, **P** The quantitative data of tumor weights (**O**) and tumor growth curves (**P**) of xenograft tumors. Error bars represent mean ± SD. * means *p* < 0.05, ** means *p* < 0.01, *** means *p* < 0.001, **** means *p* < 0.0001

Among the MCI-included malignant genes, UQCRFS1 has been documented to possess the vital involvement in tumor progression [46–48]. However, its role in TNBC remains elusive. Therefore, we decided to explore the expression and clinical significance of UQCRFS1 in TNBC. Initially, the expression of UQCRFS1 was compared between the paired non-tumoral and TNBC samples in GSE76250 dataset, which displayed that UQCRFS1 was apparently enhanced in TNBC tissues (Fig. 9A). Subsequently, qRT-PCR analysis was utilized to validate UQCRFS1 mRNA expression in cell lines, observing that UQCRFS1 mRNA expression was also elevated in TNBC cell lines compared to the mammary epithelial cell line MCF-10A (Fig. 9B), which was consistent with the results of previous bioinformatic analyses. Additionally, the FUSCC proteome data was retrieved, which embodied the proteomic information of TNBC patients, hinting that the protein expression of UQCRFS1 was similarly upregulated in TNBC tissues (Fig. 9C). Western blot analysis confirmed that UQCRFS1 was significantly enhanced in TNBC cell lines at the protein level (Fig. 9D). Then, we collected a ST dataset containing a TNBC patient, which delineated that UQCRFS1 was mainly expressed in tumor areas rather than normal duct area (Fig. 9E, F).

### UQCRFS1 accelerates the proliferation and migration of TNBC cells in vitro and tumor growth in vivo

Furthermore, to dig out the functional effect of UQCRFS1 to TNBC cells, a series of in vitro and in vivo functional experimental were implemented under the situation of UQCRFS1 knockdown in MDA-MB-231 and BT549 cells, which was carried out utilizing two siRNAs (Fig. 9G). Then, CCK-8 assays revealed that knockdown of UQCRFS1 significantly restrained TNBC cells proliferation (Fig. 9H). Afterwards, Transwell and colony formation assays were conducted to unravel whether UQCRFS1 exerted effects to the capacity of migration and colony formation of TNBC cells in vitro. Notably, UQCRFS1 knockdown essentially curbed TNBC cells migration and colony formation (Fig. 9I–K). In order to investigate the impact of UQCRFS1 on tumor growth in vivo, xenograft models were created utilizing MDA-MB-231 and BT549 cells that undergoing stable UQCRFS1 knockdown and their corresponding control cells (Fig. 9L), which delving that the UQCRFS1 knockdown enabled to hamper the tumor weight and growth curves of xenograft tumors compared to the control group (Fig. 9M–P). Additionally, we overexpressed UQCRFS1 plasmid in MDA-MB-231 cell stably knocking down UQCRFS1 and subsequently performed CCK-8 and transwell assays in vitro as well as xenograft tumor models in nude mice in vivo. Initially, we validated the protein levels of UQCRFS1 in co-transfected cells and found that exogenous UQCRFS1-overexpressing plasmid (UQCRFS1-OE) could significantly recover the protein level of UQCRFS1 in MDA-MB-231 stably knocking down UQCRFS1 (shUQCRFS1) (Fig. S2A). The results of CCK-8 and transwell assays demonstrated that the restoration of UQCRFS1 expression obviously promoted the impaired proliferative and migrative ability induced by knocking down UQCRFS1 in MDA-MB-231 cells (Fig. S2B-C). Besides, the result of xenograft tumor models in nude mice illustrated that recovering UQCRFS1 expression visibly enhanced the tumor weight in MDA-MB-231 cells (Fig. S2D-E). To summarize, these results unraveled that UQCRFS1 potentiated TNBC cells proliferation and migration in vitro and in vivo, enlightening that UQCRFS1 might be a promising biomarker in TNBC.

### High expression of UQCRFS1 is correlated with immunosuppressive TME and non-responsiveness to immune checkpoint blockers in TNBC

To unravel the connection of UQCRFS1 and TME heterogeneity, we evaluated the differences of the infiltrating TME cells between TNBC patients of METABRIC dataset with the high- and low-expression of UQCRFS1 utilizing ESTIMATE and CIBERSORT algorithms. The result of ESTIMATE algorithm uncovered that TNBC patients with the high-expression of UQCRFS1 gained less immune cell infiltration than those with low-expression of UQCRFS1, whereas the infiltration of the stromal or malignant cells differed slightly between two groups (Fig. S3A). Besides, the result of CIBERSORT algorithm illustrated that several immune-activating cell types, including CD8^+^ T, follicular helper T, activated NK, and M1 macrophages, were significantly decreased in TNBC patients with the high-expression of UQCRFS1 when compared to the low-expression group (Fig. S3B). The above results hinted that the high expression of UQCRFS1 was significantly associated with the immunosuppressive TME. Furthermore, to explore the correlation between UQCRFS1 and the responsiveness to immune checkpoint blockers in TNBC, we compared the expression levels of UQCRFS1 in nCR and pCR groups of GSE173839 dataset, and demonstrated that the expression level of UQCRFS1 in nCR group was obviously enhanced in comparison to pCR group (Fig. S3C), which implied that the high-expression of UQCRFS1 was closely correlated with the resistance to immune checkpoint blockers in TNBC.

## Discussion

The inherent intra-tumoral heterogeneity of TNBC has been considered as a contributing factor to its poor survival rate and lack of effective treatment options. Numerous studies have endeavored to discern the discrete sub-populations and investigate the underlying mechanisms of the existed cells of TME in disease carcinogenesis, as well as develop strategies for the treatment of various cancers employing scRNA-seq [49–51]. Nevertheless, the gene expression profiles of various gene clusters in TNBC remain intricate. Hence, there is a strong need to investigate the heterogeneity of TNBC and elucidate the fundamental mechanisms in enhancing TNBC prognosis.

In the present research, the comprehensive analyses were executed on our self-tested single-cell RNA sequencing dataset which consisted of TNBC treatment-naïve patients, and identified eight major cell types, namely B cells, endothelial cells, epithelial cells, fibroblasts, mural cells, myeloid cells, plasma cells, and T cells. Additionally, the distinct subclusters in each major cell type were further dug out, which displayed diverse characteristics. In detail, myeloid cells were reclustered into six subclusters, and we focused on the macrophages including S100A8^+^ Macro, IL1B^+^ Macro, FOLR2^+^ Macro, and MKI67^+^ Macro. Previous studies have demonstrated that macrophages possess the ability to facilitate the development of tumors by activating the TGF-β signaling pathway within tumor cells [52–54]. In our study, we corroborated that hallmark terms associated with carcinogenesis such as TGF_BETA_SIGNALING, P53_PATHWAY, and WNT_BETA _CATENIN_SIGNALING, were enriched in IL1B^+^ Macro subcluster. Moreover, plenty of TFs were activated in IL1B^+^ Macro subcluster. Thus, we deduced that IL1B^+^ Macro constituted a larger percentage of the macrophage population in TNBC, and there was a possibility of the presence of intercommunication among TGF-β, p53 and Wnt/β-catenin pathways which could potentially impact the progression of TNBC. Of note, Jang et al. demonstrated that the soluble CD44 antigen in the cell membrane of TNBC cells triggered IL1B production of Macro and thereby promoted the progression of TNBC [55], which hinted the importance of IL1B^+^ Macro subcluster in TNBC progression. Fibroblasts might interact with the TME and induce EMT, contributing to cancer biology [56, 57]. Our study delineated that Fib_C0 and Fib_C1 possessed the highly expressed EMT and ECM-related genes, like biglycan (BGN) and MMPs. Additionally, the previous study unraveled that biglycan derived from the cancer-associated fibroblasts served as a potential therapeutic target in immunotherapy resistance in TNBC [58]. Afterwards, the distinguishment of T cells assisted of us to appreciate the correlation between IL32^high^ Treg and tumor escape, which demonstrated that TNBC patients with more IL32^high^ Treg infiltration probably gained the worsened prognosis. Likewise, IL32 expression in Treg was identified to correlate with the impaired function of CD8^+^ T cell and tumor progression [59, 60], in line with our investigations.

Aiming to dig out the clinical utility of differentially expressed markers in malignant epithelial cells, we developed an MCI model comprising five genes (BGN, SDC1, IMPDH2, SPINT1, and UQCRFS1) that exhibited promising prognostic significance in predicting survival outcomes in TNBC. In recent years, numerous signatures have been verified for prognostic prediction using bulk transcription datasets [61–64]. Nonetheless, the researchers failed to consider the heterogeneity of tumors. Consequently, the generation of MCI through the activation of malignant genes at the single-cell level was proved to be more reliable in this study, which showed the robust excellent efficacy in predicting TNBC patients’ prognoses among four independent cohorts. Besides, our result unveiled that MCI turned to be an independent prognostic factor as well as a reliable indicator for immunotherapy resistance. Mechanistically, it was reported that BGN secreted by cancer cells was elicited by Akt/mTOR and MNK/eIF4E pathways and offered the immunosuppressive microenvironment to cancer cells by recruiting suppressive myeloid cells [65]. Similarly, syndecan-1 (SDC1) restrained IFN-γ-STAT1 signaling and antigen presentation in tumor cells and thereby attenuated the sensitivity to T cell-mediated cytotoxicity and induced immunotherapy resistance [66]. What’s more, there have been several previous studies exploring the effects of MCI-included genes in development of breast cancer. For instance, Zheng et al. found that BGN-encoding protein biglycan in cancer-associated fibroblasts was a protumor and immunosuppressive factor in TNBC, which was negatively correlated with CD8^+^ T cells [56]. Additionally, Zhong et al. demonstrated that SDC1 was highly expressed in TNBC patients and promoted the migration of MDA-MB-231 cells through a TGFb1-Smad and E-cadherin-dependent mechanism [67]. Besides, da Silva Fernandes et al. illustrated that the elevated IMPDH2 expression was associated with a worse OS in TNBC patients and enhanced the pro-tumorigenic phenotypes and doxorubicin-resistance in MDA-MB-231 cells [68]. Wu et al. found that SPINT1 was upregulated in breast cancer and relatively higher in HER-2-enriched and node positive patients, and the functional enrichment analysis showed that the co-expressed SPINT1 and SPINT2 expression were primarily involved in modulating cell attachment and migration [69]. These previous studies hinted that our prognostic model MCI indeed predicted the progression of TNBC patients.

Furthermore, UQCRFS1, a crucial gene in MCI model, is one of the subunits that make up the cytochrome-C oxidoreductase complex, a part of the mitochondrial respiratory chain responsible for transferring electrons from ubiquinone to cytochrome-C [70]. UQCRFS1 has been clarified to play an important role in mitochondrial diseases and a variety of cancers, including ovarian tumor [71], gastric cancer [72], and Cutaneous Melanoma [73]. Another study has obtained samples from image-guided core needle biopsies in 40 patients with untreated BC and used fluorescent in situ hybridization to investigate UQCRFS1 gene amplification, and the results implied that UQCRFS1 was significantly upregulated in the development of a more aggressive phenotype of BC [74]. Of vital importance, we firstly used public datasets to confirm that both the mRNA and protein levels of UQCRFS1 were higher in TNBC tissues compared with normal tissues in the present research. Additionally, the function of UQCRFS1 was verified by in vivo and in vitro experiments, which depicted that knockdown of UQCRFS1 significantly inhibited the proliferative and migratory capacities of TNBC cells in vivo and in vitro. What’s more, the connection of UQCRFS1 expression and TME heterogeneity was explored, which implied that the high-expression of UQCRFS1 was closely correlated with the immunosuppressive TME and the resistance to immune checkpoint blockers in TNBC. Collectively, the results above signified that UQCRFS1 turned to be a potential biomarker for TNBC. However, the underlying mechanism of UQCRFS1 in tumor progression and immunotherapy resistance should be further investigated in the future research, which will enhance the utilization of UQCRFS1 in clinical practices.

## Conclusion

Conclusively, our study offers innovative perspectives on comprehending the heterogeneity within TME cells of TNBC, thereby facilitating the elucidation of TNBC biology and providing clinical recommendations for TNBC patients’ prognosis. Specially, IL32^high^ Treg infiltration was ascertained to associate with tumor evasion and the detrimental prognosis of TNBC patients. Additionally, MCI model constructed on the basis of the malignant-related genes enabled to predict the worsened prognosis and immunotherapy resistance of TNBC patients. Ultimately, UQCRFS1, one of crucial genes in MCI model, was deciphered to be a deleterious factor in tumor progression and a potential target in TNBC treatment.

## Supplementary Information

Supplementary material 1.

## Data Availability

All data generated or analyzed during this study are included in this article. The raw scRNA sequencing data in this study have been deposited in the Genome Sequence Archive in National Genomics Data Center, China National Center for Bioinformation/Beijing Institute of Genomics, Chinese Academy of Sciences (GSA: HRA008417) that are publicly accessible at https://ngdc.cncb.ac.cn/gsa.

## Abbreviations

TNBC: Triple-negative breast cancer
TME: Tumor immune microenvironment
TF: Transcription factor
LASSO: Least Absolute Shrinkage and Selection Operator
MCI: Malignant cell index
UQCRFS1: Ubiquinol-Cytochrome C Reductase, Rieske Iron-Sulfur Polypeptide 1
scRNA-seq: Single-cell RNA sequencing
qRT-PCR: Quantitative Real-time Polymerase Chain Reaction
CNV: Copy number variation
ST: Spatial transcriptome
HGV: Highly variable gene
tSNE: T-distributed stochastic neighbor embedding algorithm
UMAP: Uniform manifold approximation and projection algorithm
PCA: Principal component analysis
DCA: Decision curve analysis
ATCC: American Type Culture Collection
siRNA: Small interfering RNA
shRNA: Short hairpin RNA
CCK-8: Cell Counting Kit-8
FBS: Fetal bovine serum
tDC: Tolerogenic dendritic cell
pDC: Plasmacytoid DC
Macro: Macrophage
K-M: Kaplan–Meier
ECM: Extracellular matrix
MMP: Matrix metalloproteinases
NK cell: Natural killer cell
Treg: Regulatory T cell
EMT: Epithelial mesenchymal transition
ICB: Immune checkpoint blockade
nCR: Non- Pathologic complete response
pCR: Pathologic complete response
AUC: Area under the ROC curve
BGN: Biglycan
