# Supplementary material for: Multi-omics analyses of the heterogenous immune microenvironment in triple-negative breast cancer implicate UQCRFS1 potentiates tumor progression
Source: Exp Hematol Oncol. 2025 Jun 16;14:85. doi: 10.1186/s40164-025-00672-1 (PMC12172350; doi:10.1186/s40164-025-00672-1)
Supplement: Supplementary file 1 — Supplementary material 1. [file 40164_2025_672_MOESM1_ESM.docx]

Supplementary Figures

**Multi-omics analyses of the heterogenous immune microenvironment in triple-negative breast cancer implicate UQCRFS1 potentiates tumor progression**

Yuhui Tang^1,†^, Aiqi Xu^2,†^, Zhongbiao Xu^3,†^, Jindong Xie^1,†^, Wei Huang^3^, Liulu Zhang^2^, Yitian Chen^2^, Lu Yang^3,*^, Shasha Du^3,*^, Kun Wang^2,*^

^1^ State Key Laboratory of Oncology in South China, Guangdong Provincial Clinical Research Center for Cancer, Sun Yat-sen University Cancer Center, Guangzhou 510060, P. R. China.

^2^ Department of Breast Cancer, Cancer Center, Guangdong Provincial People's Hospital (Guangdong Academy of Medical Sciences), Southern Medical University, Guangzhou 510080, P. R. China.

^3^ Department of Radiotherapy, Cancer Center, Guangdong Provincial People's Hospital (Guangdong Academy of Medical Sciences), Southern Medical University, Guangzhou, 510080, P. R. China.

Figure legends


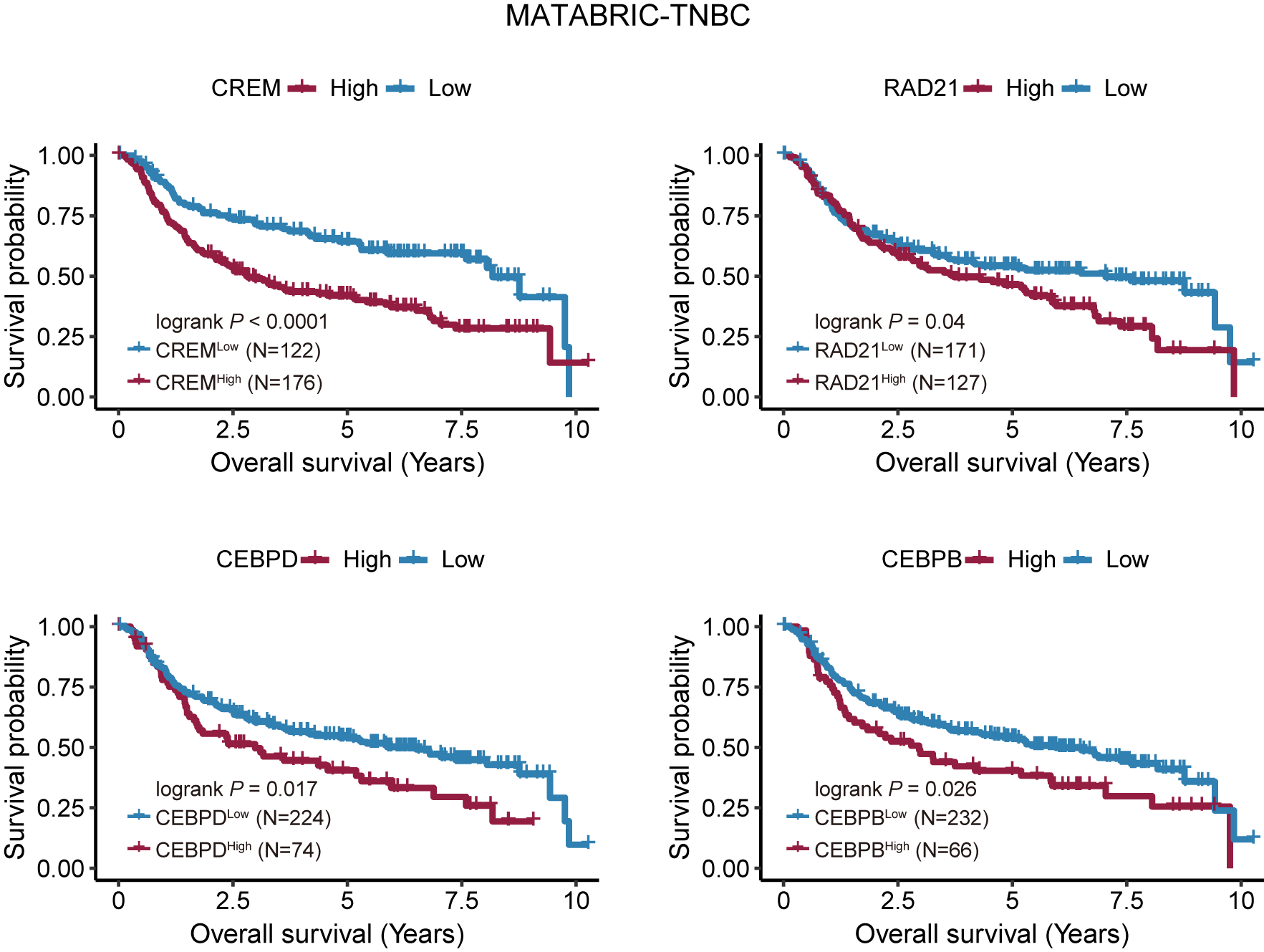


**Fig. S1**: Survival analyses of the specific TFs marking every single macrophage subcluster in GSE58812 dataset.


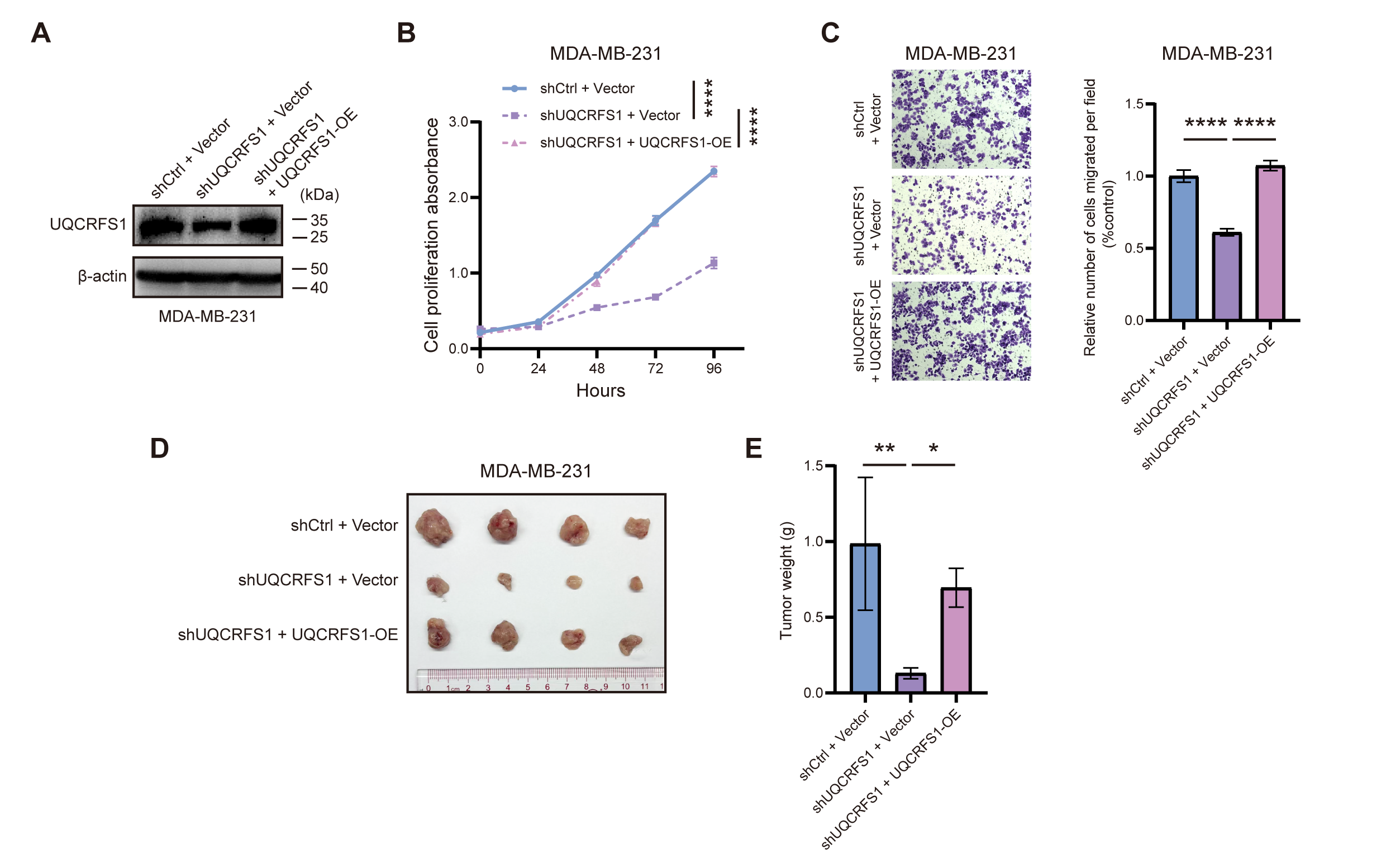


**Fig. S2**: The restoration of UQCRFS1 expression recovers the tumor progression in the UQCRFS1-silcend TNBC cells. **A** Western blotting analysis validating the protein level of UQCRFS1 in co-transfected MDA-MB-231 cells. **B** The result of CCK-8 assays in co-transfected MDA-MB-231 cells. **C** The representative images (left) and statistical data (right) of transwell assays performed in co-transfected MDA-MB-231 cells. **D** The representative images of xenograft tumor morphology using co-transfected MDA-MB-231 cells. **E** The quantitative data of tumor weights of xenograft tumors. Error bars represent mean ± SD. * means *p* < 0.05, ** means *p* < 0.01, and **** means *p* < 0.0001.


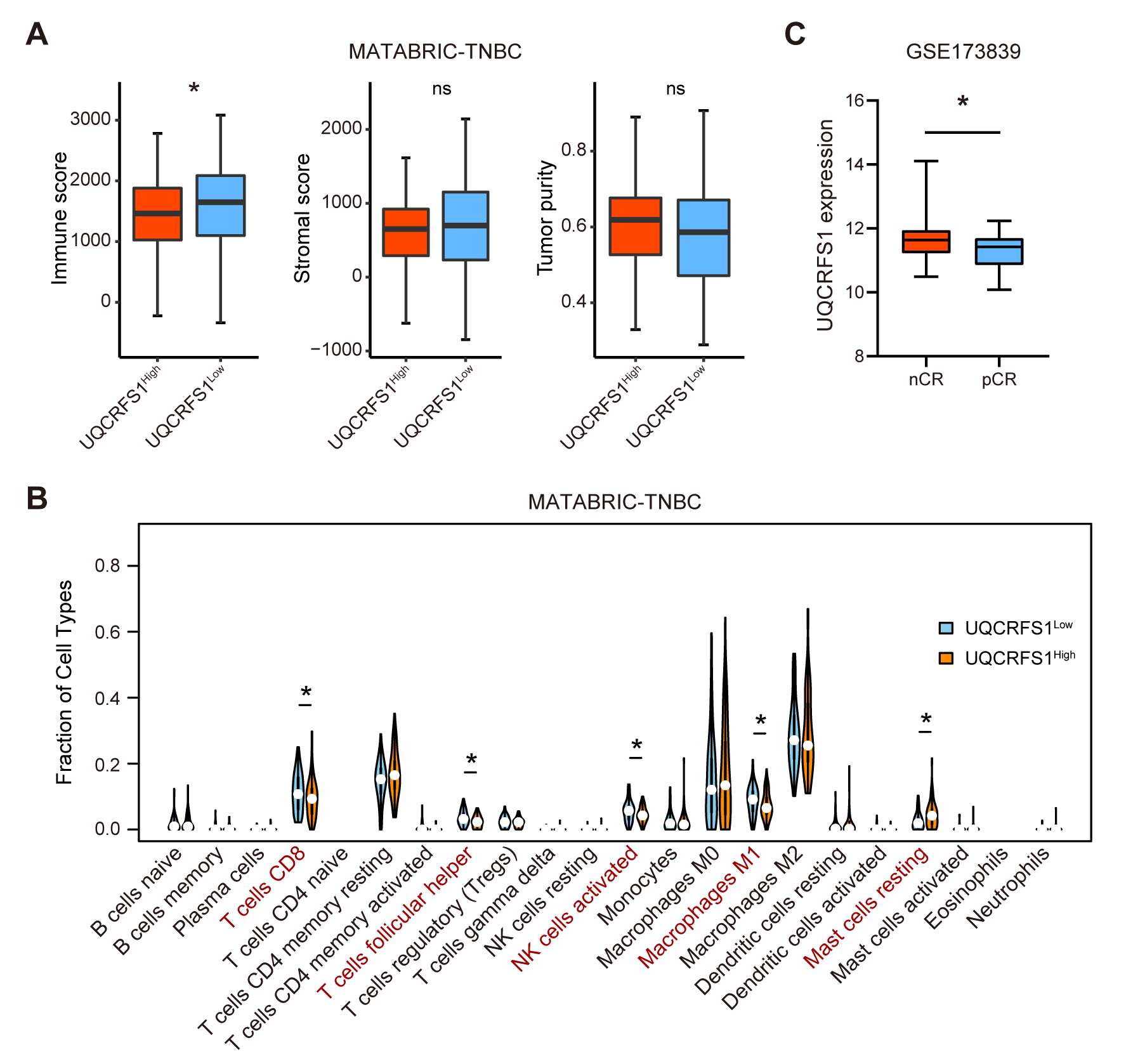


**Fig. S3**: High-expression of UQCRFS1 is correlated with immunosuppressive TME and non- responsiveness to immune checkpoint blockers in TNBC. **A** The result of ESTIMATE algorithm performed in TNBC patients of METABRIC dataset with the high- and low-expression of UQCRFS1. **B** The result of ESTIMATE algorithm utilized in TNBC patients of METABRIC dataset with the high- and low-expression of UQCRFS1. **C** The result of comparing the expression levels of UQCRFS1 in nCR and pCR groups of GSE173839. ns means not significant, and * means *p* < 0.05.

| Supplementary Table S1. Clinical information of biopsy tissues for scRNA-seq | | | | | | |
| --- | --- | --- | --- | --- | --- | --- |
| Patient index | Molecular subtype | Grade | cT | cN | Neoadjuvant therapy | Response to neoadjuvant therapy |
| P1 | TNBC | 3 | 2 | 0 | ICB therapy and chemotherapy | pCR |
| P2 | TNBC | 3 | 4 | 2 | ICB therapy and chemotherapy | Not applicable |
| P3 | TNBC | 2 | 2 | 0 | ICB therapy and chemotherapy | pCR |
| P4 | TNBC | 3 | 1 | 1 | ICB therapy and chemotherapy | pCR |
| P5 | TNBC | 3 | 2 | 0 | ICB therapy and chemotherapy | nCR |
| P6 | TNBC | 3 | 4 | 3 | ICB therapy and chemotherapy | nCR |
| P7 | TNBC | 2 | 2 | 1 | ICB therapy and chemotherapy | Not applicable |
| P8 | TNBC | 3 | 3 | 2 | ICB therapy and chemotherapy | nCR |
| P9 | TNBC | 3 | 2 | 0 | ICB therapy and chemotherapy | pCR |

| Supplementary Table S2. Sequences of the siRNAs and shRNA | |
| --- | --- |
| siCtrl | Sense: 5'-UUCUCCGAACGUGUCACGUTT  Antisense: 5’-ACGUGACACGUUCGGAGAATT |
| siUQCRFS1#1 | Sense: 5'-GCGAGGCUAGGAAAGGUUUTT  Antisense: 5’-AAACCUUUCCUAGCCUCGCTT |
| siUQCRFS1#2 | Sense: 5'- GCAGUUGAAUUAUCACAGUTT  Antisense: 5’- ACUGUGAUAAUUCAACUGCTT |
| shUQCRFS1 | 5'-GCGAGGCTAGGAAAGGTTT |

| Supplementary Table S3. Primers for qRT-PCR detection | | |
| --- | --- | --- |
| UQCRFS1 | Forward | CTGAATACCGCCGCCTTGAA |
|  | Reverse | ATGCGACACCCACAGTAGTTA |
| β-actin | Forward | CATGTACGTTGCTATCCAGGC |
|  | Reverse | CTCCTTAATGTCACGCACGAT |
